# Health-related quality of life, culture and communication: a comparative study in children with cancer in Argentina and Sweden

**DOI:** 10.1186/s41687-018-0075-0

**Published:** 2018-10-17

**Authors:** Emelie Stenmarker, Karin Mellgren, Mónica Matus, Anna Schroder Hakansson, Margaretha Stenmarker

**Affiliations:** 1 Department of Orthopaedics, Sodra Alvsborg Hospital, Bramhultsvägen 53, SE-501 82, Boras, Sweden; 2 Department of Paediatrics, Institution for Clinical Sciences, Sahlgrenska Academy, University of Gothenburg, SE- 416 85 Gothenburg, Sweden; 3 Instituto de Hematologia y Oncologia del Rosario, San Juan 2395 (2000), Rosario, Pcia Santa Fe, Argentina; 4 Department of Paediatrics, Futurum - the academy for health and care, Region Jonkoping Council, SE-551 85 Jonkoping, Sweden

**Keywords:** Health-related quality of life, Paediatric oncology, Patient-reported outcomes, Childhood cancer, culture, PedsQL

## Abstract

**Background:**

Malignant disorders in childhood are life-threatening conditions, and issues regarding the children’s health-related quality of life (HRQOL) are crucial in paediatric oncology. The overall aim of this study was to explore HRQOL in children with cancer in two countries, Argentina and Sweden, which have different cultural contexts. The specific aims were: to determine HRQOL by gender, age, diagnosis, treatment modality, time since diagnosis, and parental education/employment across cultures. Further aims were to assess the child/parent relationship in HRQOL and the influence of demographic variables in psychosocial and physical HRQOL in each country.

**Methods:**

A cross-sectional study was performed in 2014, including 58 children (24 females, 34 males) and 62 parents/guardians. The instrument, the Pediatric Quality of Life Inventory™ (PedsQL™, generic, cancer and fatigue modules), and medical records were used. The response rate was 97%.

**Results:**

The mean age of the children was 8.67 years (SD 5.1, range 2–18 years) and the mean time on treatment was 10.7 months (SD 8.7, range 1–30 months). The most common diagnosis was leukaemia (57%). In Argentina, in comparison with Sweden, a higher estimation of generic HRQOL was reported among adolescents (*p* = 0.022) and more cancer-related problems among school-age children (*p* < 0.0001). Children and parents in both countries confirmed the major problem with fatigue and multimodality therapy regimes, but lower levels of fatigue were reported in Argentina. Adolescents and children with solid tumours appeared as vulnerable groups. In Sweden, children whose mothers had post-secondary education reported less cancer-related problems (*p* = 0.031). Good relationships were found between child/parent reports in Argentina regarding the fatigue module (*p* = 0.034) and physical subscale (*p* = 0.014), and in Sweden regarding generic health (*p* = 0.004), including psychosocial (*p* = 0.006) and physical subscales (*p* = 0.042), and cancer (*p* = 0.001), and fatigue (*p* < 0.0001) modules. In Sweden, psychosocial health (OR 7.5; *p* = 0.007) and physical health (OR 6.2; *p* = 0.011) were positively influenced by being a school-age child.

**Conclusions:**

Fatigue is as a major problem across cultures. Still, being in school facilitates recovery. Good relationships in psychosocial HRQOL highlight professional challenges regarding severe issues and open communication, and the need of performing comparative studies of HRQOL of children with cancer from different cultural backgrounds.

## Background

Malignant disorders in childhood are life-threatening conditions. More than 175,300 children all over the world are diagnosed with cancer every year [1]. The causes of the majority of childhood cancers are unknown [2], and the diseases are relatively rare in children aged one to 14 years of age and account for less than 2% of all human cancers [3]. The distribution of diagnosis is approximately as follows; leukaemia (30%), central nervous system tumours (CNS) (28%), lymphomas (12%), and the rest are miscellaneous diagnoses [4]. This pattern of diagnosis is similar in North America, South America and Europe [5]. Today, if adequate and sufficient treatment is given, 80% of the children will survive [4, 6]. However, a plateau concerning survival rates after cancer treatment has been reached [4] and research into the patients’ quality of life (QOL) is becoming increasingly important. QOL and health-related quality of life (HRQOL) are multidimensional constructs with several definitions [7–9]. HRQOL is a measure of an individual’s self-perceived health status [9] and can be measured by psychometric instruments that may be generic or condition-specific [10]. The recommendation is to use both to capture an overall life perspective [10]. In childhood, the young individual’s cognitive and emotional development is crucial to consider when performing QOL studies [11, 12]. The measurements used are patient and proxy reports. Some studies report solely the results from parent/proxy versions [13, 14]. However, self-reports involving children with cancer have been found to be valid and reliable and are recommended [10, 15], and often used with children above the age of five years [10]. To reach a comprehensive evaluation, both children’s and parents’ (dyadic) perspectives need to be taken into account [16].

The literature reveals that the cancer disease and its treatment often affect all dimensions of HRQOL of the sick child and influence the life situation of the entire family [17]. Diminished HRQOL is reported at diagnosis [18], during treatment [19–22] and one year after diagnosis, although it steadily improves [18, 20]. Several medical long-term consequences of the treatment are known, including severe multi-organ health status [15, 23] with an increased risk of premature mortality [24]. Reports of long-term consequences influencing HRQOL are not conclusive; that is, the HRQOL may be reduced or equal [25–27] or even better [18, 27, 28], compared to normal distribution. According to the literature, risk factors for reduced HRQOL during treatment and in the long term are older age at diagnosis [15], female gender [20, 24, 29], being diagnosed with a CNS tumour [15, 24], increased treatment intensity, in particular cranial irradiation [15, 26], and a persistent medical condition [24]. Inconsistent results are reported regarding duration of diagnosis, i.e. reduced HRQOL related to shorter or longer [15] time since diagnosis. Family factors associated with reduced HRQOL in children with cancer have been reported, e.g. parental psychological distress [12, 15] and lower socioeconomic status [30]. Lower income and lower parental level of education may influence QOL in paediatric oncology [15, 30] and may have an impact on the child’s generic HRQOL [12]. Increased HRQOL in children has been associated with higher parental level of education [30]. On the other hand, studies have shown no significant relationship between the child’s HRQOL and socioeconomic factors, or parental educational level. However, adult childhood cancer survivors have reported poor HRQOL associated with unemployment, lack of medical insurance, lower educational attainment, and low household income [24, 26]. Variations in HRQOL in relation to culture of origin have been reported, with reduced HRQOL suggested for those of non-Caucasian ethnicity [15, 31]. In Argentina, comparative HRQOL studies of children with chronic diseases, including cancer, are asked for [31].

The overall aim of this study was to explore HRQOL in children with cancer in two countries, Argentina and Sweden, which have different cultural contexts. The specific aims were: to determine HRQOL by gender, age, diagnosis, treatment modality, time since diagnosis, and parental education/employment across cultures. Further aims were to assess child/parent relationship in HRQOL and the influence of demographic variables in psychosocial and physical HRQOL in each country.

According to the literature, we anticipated that the study population in both countries would have lower scores of HRQOL in comparison with normal distribution due to their malignant diseases [10, 11, 18, 20, 21, 30], but with no differences between countries.

## Methods

### Study design and context

The study was cross-sectional and used a validated psychometric instrument, the Pediatric Quality of Life Inventory™ (PedsQL™) [11] and medical records.

Argentina is a country with great variations in socioeconomic status, with high and low income sectors, differences in health insurance, and literacy skills [31]. In Sweden, the inhabitants have free access to health care and the social insurance system guarantees every child the same care, regardless of their parents’ socioeconomic status. Regarding school activities, studies in compulsory schools are mandatory (nine grades excluding kindergarten).

### Participants and treatment

A total of 60 children and adolescents and their parents/guardians (60 dyads) were invited to take part in the study. Half this sample size (*n* = 30 dyads) would be sufficient to detect a medium effect size for a paired *t*-test comparison of QOL scores between children and parents (alpha level 0.05 and approximately 80% power) [32]. In both countries, the diagnostic procedures as well as the oncological treatment are centralised to paediatric oncology centres and public hospitals. The therapy includes primarily cytotoxic treatment. The treatment may last for several months to 2.5 years. Depending on the specific diagnosis, surgery and radiotherapy may be given as well [4, 5].

### Data collection

The study was performed in August to September (Sweden) and October to November (Argentina), 2014. In Sweden, patients were recruited from the day care clinic/paediatric department at a childhood cancer centre, while in Argentina they were recruited from an outpatient paediatric oncology clinic and a paediatric department. Children and adolescents (ages two to18) who had been under treatment for malignant diseases for at least one month or who had completed the treatment within the last month, and their parents were included. Children who were found to be too ill to participate, had major developmental disorders or comorbidity, or were not able to speak or read Swedish or Spanish were excluded. The patients and parents were given verbal and written information about the study in Swedish (Sweden) or Spanish (Argentina) before they were asked to participate, and informed consent was obtained. All participants completed the questionnaires at the hospital or at an outpatient clinic. The questionnaires were self-administered to the eight to18 years old age group, with patients and parents separated as far as possible. ES interview-administered the questionnaires [11] in Sweden in the five to seven years age group and was present in the room with one of the authors (MM) in Argentina, but was not involved in the patients’ care. If necessary, questions were answered concerning the study and the instrument, and more verbal information was given before the forms were completed. The availability of trained interviewers was consistent with the recommendations when validating the instrument in Argentina [31]. In accordance with the PedsQL™ instructions [11], only parent proxy reports were used for children aged two to four years.

### Study instruments

Study-specific questions with demographic data were obtained from the patients, their parents/guardians, and from medical records. The questions covered the following areas: gender, age, onset of disease, diagnosis, treatment (cytotoxic treatment/immunotherapy, surgery, radiotherapy, stem cell transplantation), relapse (yes/no), education/employment (mother and fathers), and place of residence.

HRQOL was evaluated by using the internationally well-established instrument PedsQL™ [11]. This instrument was originally developed in the U.S. in English and Spanish and has shown good internal consistency, has been translated to several languages via cross-cultural language adaptation methods, and has been used for different chronic disorders and healthy children [31, 33]. PedsQL™ has been validated in Sweden [34] and Argentina [31] and in both countries the instrument has shown good feasibility [31, 34]. PedsQL™ has demonstrated good construct validity, predictive validity, and responsiveness with outpatient and inpatient paediatric patients [32]. For children with cancer, PedsQL™ is the most common self-report instrument used worldwide [8].

PedsQL™ consists of different modules and in the present study we used a generic, a disease-specific, and a fatigue scale [11]. One measurement system was created when combining these three modules [11]. The PedsQL™ 4.0 Generic Core Scale is a multidimensional questionnaire with age-matched items presented within four subscales. These subscales may be further analysed and presented as the “Psychosocial Health Summary Score” (emotional, social and school functioning) and “Physical Health Summary Score” (physical functioning). The PedsQL™ 3.0 Cancer Module is particularly designed for paediatric cancer and can be split up into eight subscales regarding pain and hurt, nausea, procedural anxiety, treatment anxiety, worry, cognitive problems, perceived physical appearance and communication. The PedsQL™ Multidimensional Fatigue Scale encompasses three subscales (general fatigue, sleep/rest fatigue and cognitive fatigue) and is used for measuring aspects of fatigue in paediatric patients [11].

The PedsQL™ scales are adjusted for children 8 to 18 years old (five to seven, eight to 12, and 13 to 18) with parent/proxy versions even for the two to four years age group [11]. Children and their parents rate, on a Likert scale, how problematic each item has been during the past one month. When analysing the completed questionnaires, HRQOL is expected to be better for those having higher scores (scale 0 to 100), which may also be expressed as having fewer problems.

Age-specific questionnaires were used for every participant and their proxy versions [11]. Due to a low number of study participants in the age groups five to seven years (*n* = 10) and eight to 12 years (*n* = 10), the material was analysed in larger categories according to previous research [12]: toddlers/pre-school-age (two to four years), school-age (five to 12 years) and adolescence (13 to 18 years).

### Statistical methods

The statistical analyses were performed using the Statistical Package for the Social Sciences version 21.0 (IBM Corp, Armonk, New York, USA). Statistical significance was attained at *p*-value < 0.05. The following statistical symbols were used: * = *p* < 0.05, ** = *p* < 0.01, *** = *p* < 0.001, and **** = *p* < 0.0001. Descriptive statistics were obtained using frequencies, mean values, medians, standard deviations and percentage. Regarding the scoring of HRQOL, Pearson’s correlation coefficient was analysed to detect potential intercorrelations between patients and parents’ reports. To compare proportions of categorical variables between groups, chi-square statistics were obtained. When more than 20% of the cells had an expected value of less than five, Fisher’s exact test was used. The Mann-Whitney test was performed in order to compare the mean values between two independent variables. When comparing two or more independent variables, the Kruskal-Wallis test was used. Finally, the generic HRQOL total scale (including subscales) was dichotomised based on the medians. Binary logistic regression analyses (method = ENTER) were performed to study potential influences of independent demographic variables on HRQOL. To quantify predictors, odds ratios (ORs) were calculated.

## Results

### Descriptive statistics

The characteristics of the study population are presented in Table 1. In all, 58 children (24 females, 34 males) and 62 parents/guardians participated. Two of the invited children (*n* = 60), one in each country, had severe side effects of the treatment/were too ill to complete the study. Proxy versions of the PedsQL™ were answered by one of the parents (*n* = 53), by mothers (*n* = 43), by fathers (*n* = 10), by both parents (n = 4), or by a grandparent (n = 1). The response rate was 97%. The mean age of children (*n* = 58) was 8.67 years (SD 5.1, range 2–18 years). In Sweden the mean age was 8.68 years (SD 5.16, range 2–18 years) and in Argentina 8.67 years (SD 5.18, range 2–18 years). Their median age was 7.0 years and 8.0 years, respectively. The mean time on treatment was 10.7 months (SD 8.7, range 1–30 months) for the entire body of material ((Sweden 10.5 months (SD 9.5, range 1–30 months), Argentina 10.9 months (SD 8.1, range 1–30 months)). One participant answered the questionnaires after one month of being off treatment. During the study period four children (6.9%) relapsed and died, two in each country.

**Table 1:** Demographic data and clinical characteristics of participating children and parents

Characteristics	Participants	X^2^ between countries’^1^	Argentina	X^2^ within Argentina^a^	Sweden	X^2^ within Sweden^a^
Total number, n (%)	58		30 (51.7)		28 (48.3)	
Gender distribution, n (%)		0.45^1^		0.20^1^		0.85^1^
Female	24 (41.4)		11 (36.7)		13 (46.4)	
Male	34 (58.6)		19 (63.3)		15 (53.6)	
Age groups, mean (sd)	8.67 (5.1)	0.80^1^	8.67(5.18)	1.00^1^	8.68 (5.16)	1.00^1^
2–4 years	19 (32.8)		10 (33.3)		9 (32.1)	
5–7 years	10 (17.2)		4 (13.3)		6 (21.4)	
8–12 years	10 (17.2)		6 (20.0)		4 (14.3)	
13–18 years	19 (32.8)		10 (33.3)		9 (32.1)	
Diagnostic groups, n (%)		0.11^1^		***p*** **< 0.0001**		0.24^1^
I Leukaemia	33 (56.9)		21 (70.0)	***p*** **= 0.004**	12 (42.9)	
II Solid tumours	17 (29.3)		6 (20.0)	I-II	11 (39.3)	
III CNS tumours	8(13.8)		3 (10.0)	***p*** **< 0.001** I-III	5 (17.9)	
Treatment modality, n (%)		0.12^1^		***p*** **< 0.0001**		0.35^1^
I Chemotherapy	42 (72.4)		25 (83.3)	***p*** **< 0.0001** I-II	17 (60.7)	
II Combined therapy	16 (27.6)		5 (16.7)		11 (39.3)	
Time since diagnosis (months), mean ^(sd)^	10.7 (8.7)	0.67^1^	10.9 (8.1)		10.5(9.5)	
Completed questionnaires, n (%)		0.77^1^		***p*** **< 0.0001**		***p*** **< 0.0001**
I Female (F)	43 (74.1)		23 (76.7)	***p*** **< 0.0001** I-II	20 (71.4)	***p*** **< 0.0001** I-II
II Male (M)	10 (17.2)		5 (16.7)		5 (17.9)	
Both parents	4 (6.9)		2 (6.7)		2 (7.1)	
Grandparent	1 (1.7)		0		1 (3.6)	
Parents/Couples employment, n (%)	116	***p*** **< 0.0001**	60 (51.7)	***p*** **< 0.0001**	56 (48.3)	***p*** **= 0.045**
I Work tasks; post-secondary education	41 (35.3)		16 (26.7)		25 (44.6)	***p*** **= 0.045** I-II (F)
						***p*** **= 0.012** I-III (F)
II Work tasks; no post-secondary education	39 (33.6)		17 (28.3)		22 (39.3)	
III Unemployed	32 (27.6)	***p*** **< 0.0001** I-III (F)	24 (40.0)	***p*** **= 0.005** I-III (F)	8 (14.3)	
				***p*** **< 0.001** II-III (F)		
		***p*** **< 0.0001** I-II (F)		***p*** **= 0.048** II-III (M)		
Not stated	4 (3.4)		3(5.0)		1(18)	

^a^Tested for difference in proportions by %2 test or in cases of small numbers, Fisher’s exact test. Significant differences are highlighted in bold type, ^1^Non significant *F* female, *M* male

### Diagnoses and treatment

The diagnoses were divided into three diagnostic groups. The most common diagnosis was leukaemia (57%). One patient with Langerhans Cell Histiocytosis was included in this group. The diagnostic group with solid tumours (29%) included lymphomas, liver tumours, renal tumours, soft tissue sarcoma and one neuroblastoma. The third group (14%) was for CNS tumours. The children received different treatment modalities including chemotherapy, surgery and radiation therapy. The material was analysed, comparing cytotoxic treatment solely and/or in combination with surgery and/or radiotherapy.

### Parental education/employment

The participating parent stated his/her and the partner’s (*n* = 116) profession. In total, 78 professions were represented. Occupational data were classified into three groups: work tasks that required post-secondary education (35%), work tasks that did not require post-secondary education (34%), and a group of parents/guardians who did not work (28%).

### Demographic variables (Table 1)

Argentina and Sweden: No significant differences were found across countries when comparing children’s gender and age distribution, diagnostic groups, time since diagnosis, or parental gender distribution. In both countries there was an overrepresentation of participating mothers compared to fathers. In Argentina more mothers were unemployed compared to Sweden.

Argentina: Children diagnosed with leukaemia were overrepresented compared to children diagnosed with solid tumours and CNS tumours, and more children were treated with cytotoxic treatment solely. Focusing on parents’ education, more mothers and fathers were unemployed in comparison with the group who had work tasks that did not require post-secondary education, and regarding mothers, even in comparison with the group who had work tasks that required post-secondary education.

Sweden: Mothers who had post-secondary education were overrepresented in comparison with the other education/employment groups.

### Relationships between children’s and parents’ versions

Within each country a calculation of potential variable relation (correlation) was performed. Good agreement between child self-report and parent proxy-version was defined as *r* > 0.50 and poor agreement as *r* < 0.30 [14]. In Sweden, the generic module (*r* = 0.632**, *p* = 0.004) including psychosocial health (*r* = 0.605**, *p* = 0.006), physical health (*r* = 0.471*, *p* = 0.042), and the cancer (*r* = 0.703**, *p* = 0.001) and fatigue modules (*r* = 0.725**, *p* < 0.0001) were significant. In Argentina, the generic physical scale (*r* = 0.551*, *p* = 0.014) and the fatigue module were significant (*r* = 0.489*, *p* = 0.034), but the total generic rating (*r* = 0.422, *p* = 0.072), the psychosocial health scale (*r* = 0.171, *p* = 0.484) and the cancer module (*r* = 0.289, *p* = 0.231) were not significant.

### The pediatric quality of life inventory™ (PedsQL™)

PedsQL™ data (mean scores, standard deviations, and *p*-values) are presented between the two countries and within each country (Tables 2, 3, 4, 5, 6).

**Table 2:** PedsQL™, children’s self-reports: comparison between Argentina and Sweden

	Generic Core Scale		*p*-value	Cancer Module		*p*-value	Multidimensional Fatigue Module		*p*-value
	Argentina mean (sd)	Sweden mean (sd)		Argentina mean (sd)	Sweden mean (sd)		Argentina mean (sd)	Sweden mean (sd)	
Gender									
Female	68.8(11.0)	65.3(19.3)	0.58	69.6(12.1)	74.7(15.6)	0.30	77.1(14.2)	63.8(15.4)	0.07
Male	66.0(13.3)	62.2(12.3)	0.96	70.4(11.2)	67.9(22.6)	0.06	75.5(13.8)	56.7(16.8)	**0.01**
Age groups									
5 to 12 years	70.9(11.3)	78.3(11.9)	0.20	66.8(12.2)	85.5(5.9)	***p*** **< 0.0001**	77.9(14.4)	69.8(11.5)	0.17
13 to 18 years	63.5(12.6)	47.6(15.9)	**0.02**	73.1(9.9)	56.0(18.1)	0.08	74.4(13.3)	50.0(14.1)	**0.002**
Diagnostic groups									
Leukaemia	68.4(12.3)	70.4(20.8)	0.05	73.2(8.7)	80.6(22.1)	0.06	77.0(13.5)	65.7(16.7)	0.17
Solid tumours	66.7(15.3)	56.4(18.2)	0.36	70.6(7.7)	64.3(19.6)	0.79	79.1(15.1)	51.9(12.0)	**0.009**
CNS tumours	59.8(1.5)	75.5(25.8)	0.80	50.5(17.7)	77.4(5.9)	0.20	63.2(6.2)	78.3(5.8)	0.10
Treatment modality									
Chemotherapy	70.0(12.4)	60.8(21.5)	0.24	73.8(8.3)	70.8(24.3)	0.70	80.7(11.4)	57.3(18.6)	**0.003**
Combined therapy	58.7(7.8)	67.2(20.4)	0.58	59.6(12.7)	72.4(14.8)	0.06	63.2(11.3)	64.0(12.7)	0.92

Note: Scores range from 0 to 100; higher scores represent a better HRQOL. Significant differences are highlighted in bold type

*sd* standard deviation

**Table 3:** PedsQL ™, parents’/proxy self-reports: comparison between Argentina and Sweden

Characteristiscs	Generic Core Scale		*p*-value	Cancer Module		*p*-value	Multidimensional Fatigue Module		*p*-value
	Argentina mean (sd)	Sweden mean (sd)		Argentina mean (sd)	Sweden mean (sd)		Argentina mean (sd)	Sweden mean (sd)	
Gender									
Female	55.5(16.6)	59.2(19.0)	0.72	68.6(12.2)	67.7(15.8)	0.88	72.8(22.4)	68.2(15.5)	0.23
Male	66.9(16.4)	58.8(16.6)	0.18	66.6(14.2)	63.1(18.5)	0.59	77.9(17.7)	58.4(17.3)	**0.002**
Age groups									
2 to 4 years	69.6(21.7)	65.0(19.6)	0.51	67.9(13.6)	72.3(17.4)	0.60	77.3(26.5)	68.1(17.6)	0.14
5 to 12 years	58.1(15.4)	62.2(17.1)	0.52	66.9(13.6)	67.9(14.0)	0.97	78.6(19.1)	70.3(12.5)	0.15
13 to 18 years	60.6(12.6)	49.5(12.4)	0.09	67.3(14.1)	55.2(17.0)	0.18	72.4(10.7)	49.6(13.6)	***p*** **< 0.0001**
Diagnostic groups									
Leukaemia	63.8(16.8)	66.6(19.8)	0.69	69.3(13.9)	75.9(15.9)	0.22	73.8(14.8)	77.1(18.9)	0.33
Solid tumours	63.1(22.6)	52.3(14.1)	0.57	62.3(9.3)	56.1(15.8)	0.46	83.1(7.0)	52.2(15.2)	**0.003**
CNS tumours	54.7(10.4)	55.6(12.7)	1.00	63.7(16.6)	59.9(11.7)	0.79	54.6(16.1)	59.7(9.4)	1.00
Treatment modality									
Chemotherapy	65.8(17.1)	62.6(18.3)	0.60	69.0(13.1)	70.4(18.6)	0.53	79.3(18.5)	65.7(18.2)	**0.009**
Combined therapy	47.4(4.2)	53.5(15.1)	0.23	59.3(12.4)	57.3(11.1)	0.83	59.7(15.7)	58.7(14.5)	0.76

Note: Scores range from 0 to 100; higher scores represent a better HRQOL. Significant differences are highlighted in bold type. *sd* standard deviation

**Table 4:** PedsQL™, children’s and parents’/proxy- self-reports within Argentina

Characteristics		PedsQL (children’s score)							PedsQL (parents’ score)			
	Generic mean (sd)	*p*-value	Cancer mean (sd)	*p*-value	Fatigue mean (sd)	*p*-value	Generic mean (sd)	*p*-value	Cancer mean (sd)	*p*-value	Fatigue mean (sd)	*p*-value
Gender distribution												
Female	68.8(11.0)	0.73	69.6(12.1)	0.17	77.1(14.2)	0.98	55.5(16.6)	0.09	68.6(12.2)	0.58	72.8(22.4)	0.57
Male	66.0(13.3)		70.4(11.2)		75.5(13.8)		66.9(16.4)		66.6(14.2)		77.9(17.7)	
Age groups												
2 to 4 years							69.6(21.7)		67.9(13.6)		77.3(26.5)	
5 to 12 years	70.9(11.3)	0.13	66.8(12.2)	0.34	77.9(14.4)	0.68	58.1(15.4)	0.52	66.9(13.6)	0.99	78.6(19.1)	0.40
13 to 18 years	63.5(12.6)		73.1(9.9)		74.4(13.3)		60.7(12.6)		67.3(14.1)		72.4(10.7)	
Diagnostic groups												
Leukaemia	68.4(12.3)	0.63	73.2(8.7)	0.11	77.0(13.5)	0.36	63.8(16.8)	0.68	69.3(13.9)	0.37	77.1(18.9)	0.08
Solid tumours	66.7(15.3)		70.6(7.7)		79.1(15.1)		63.1(22.6)		62.3(9.3)		83.1(7.0)	
CNS tumours	59.8(1.5)		50.5(17.7)		63.2(6.2)		54.7(10.4)		63.7(16.6)		54.6(16.1)	
Treatment modality												
Chemotherapy	70.4(12.4)	0.09	73.8(8.3)	**0.01**	80.7(11.4)	**0.01**	65.8(17.1)	**0.04**	69.0(13.1)	0.12	79.2(18.5)	**0.02**
Combined therapy	58.7(7.8)		59.6(12.7)		63.2(11.3)		47.4(4.2)		59.3(12.4)		59.7(15.7)	

Note: Scores range from 0 to 100; higher scores represent a better HRQOL. Statistics are tests for within group differences and significant differences are highlighted in bold type. *sd* standard deviation

**Table 5:** PedsQL™, children’s and parents’/proxy self-reports within Sweden

Characteristics			PedsQL (children’s score)						PedsQL (parents’ score)			
Gender distribution	Generic mean (sd)	*p*-value	Cancer mean (sd)	*p*-value	Fatigue mean (sd)	*p*-value	Generic mean (sd)	*p*-value	Cancer mean (sd)	*p*-value	Fatigue mean (sd)	*p*-value
Female	65.3(19.3)	0.92	74.7(15.6)	0.48	63.8(15.4)	0.32	59.2 (19.0)	0.85	67.7 (15.8)	0.43	68.2 (15.5)	0.26
Male	62.2(23.3)		67.9(22.6)		56.7(16.8)		58.8 (16.6)		63.1 (18.5)		58.4 (17.3)	
Age groups												
2 to 4 years							65.0 (19.6)		72.3 (17.4)		68.1 (17.6)	
5 to 12 years	78.3(11.9)	***p*** **< 0.0001**	85.5(5.9)	***p*** **< 0.0001**	77.9(14.4)	**0.008**	62.2 (17.1)	0.14	67.9 (14.0)	0.12	70.3 (12.5)	**0.009**
13 to 18 years	47.8(15.9)		56.0(18.1)		50.0(14.1)		49.5 (12.4)		55.2 (17.0)		49.6 (13.6)	
Diagnostic groups												
Leukaemia	70.4(20.8)	0.18	80.6(22.1)	0.06	65.7(16.7)	**0.009**	66.6 (19.8)	0.24	75.9 (15.0) 56.1 (15.8)	**0.007**	73.8 (14.8)	**0.009**
Solid tumours Central nervous system	56.4(18.2)		64.3(19.6)		51.9(12.0)		52.3 (14.1)		59.9 (11.7)		52.2 (15.2)	
(CNS) tumours	75.5(25.8)		77.4(5.9)		78.3(5.8)		55.6 (12.7)		75.9 (15.0) 56.1 (15.8)		59.7 (9.4)	
Treatment modality												
Chemotherapy	60.8(21.5)	0.54	70.8(24.3)	0.54	57.3(18.6)	0.48	62.6 (18.3)	0.37	70.4 (18.6)	**0.02**	65.7 (18.2)	0.64
Combined therapy	67.2(20.4)		72.4(14.8)		64.0(12.7)		53.5 (15.1)		57.3 (11.1)		58.7 (14.5)	

Note: Scores range from 0 to 100; higher scores represent a better HRQOL. Statistics are tests for within group differences and significant differences are highlighted in bold type. sd standard deviation

**Table 6:** Comparison of parents’ education/work tasks and HRQOL in Argentina and Sweden

	Argentina mean (sd)	Sweden mean (sd)	*p*-value
Children whose parents’ have:			
	Cancer module		
Post-secondary education (F)	67.8 (4.7)	78.3 (11.8)	0.031
	Multidimensional fatigue module		
No post-secondary education (M)	76.2 (13.6)	61.4 (11.8)	0.022
Unemployed parent (F)	75.4 (18.8)	54.0 (21.0)	0.046
Parents who have:			
	Multidimensional fatigue module		
No post-secondary education (M)	71.6 (15.6)	60.3 (17.4)	0.042
Unemployed parent (F)	75.7 (16.5)	53.5 (9.2)	0.013
Unemployed parent (M)	83.3 (14.7)	56.1 (12.6)	0.031
	Generic Core Scale		
Unemployed parent (M)	76.9 (17.5)	50.3 (12.5)	0.032

Note: Scores range from 0 to 100; higher scores represent a better HRQOL

*F* female, *M* male

### Comparison of children’s self-reports in Argentina and Sweden (Table 2)

In Argentina, a higher estimation of generic HRQOL was found in the 13 to 18 years age group, and school-age children rated their cancer-linked HRQOL lower compared to the corresponding age groups in Sweden. Reduced fatigue levels were found in Argentina among males, adolescents, in the diagnostic group with solid tumours, and patients receiving cytotoxic treatment solely.

### Comparison of parents’ self-reports in Argentina and Sweden (Table 3)

In Argentina, parents reported reduced fatigue levels among males, adolescents, in the diagnostic group with solid tumours, and patients receiving cytotoxic treatment solely.

### Children’s and parents’ self-reports within Argentina (Table 4)

Children treated solely with chemotherapy rated their cancer-linked HRQOL higher and their fatigue levels lower, compared to children receiving combination therapy. Parents whose children received chemotherapy rated their child’s generic HRQOL higher and fatigue levels lower if their child was treated only with chemotherapy.

### Children’s and parents’ self-reports within Sweden (Table 5)

A lower general HRQOL rating and lower cancer-linked HRQOL, as well as more problems related to fatigue were found among adolescents, compared to the school-age group. Children with solid tumours reported higher fatigue levels compared to children with CNS tumours. Parents estimated higher cancer-linked HRQOL if their child received chemotherapy solely, in comparison with combined therapy. Parents reported more fatigue-related problems among adolescents in comparison with toddlers and school children. Higher levels of fatigue were also found when comparing diagnostic groups, i.e. parents of children with solid tumours and CNS tumours compared to parents of children with leukaemia.

### Comparison of parents’ education/work tasks and HRQOL in Argentina and Sweden (Table 6)

In Argentina, fathers who had no post-secondary education and parents who were unemployed rated lower levels of fatigue. Furthermore, children whose fathers had no post-secondary education and whose mothers were unemployed reported lower levels of fatigue. In Sweden, reduced generic HRQOL was reported by unemployed fathers. Children whose mothers had post-secondary education reported less cancer-related problems. These significant differences are presented in Table 6. No significant differences were found within each country.

### Logistic regression analyses

The dependent PedsQL™ 4.0 Generic Core Scale (total score), including the psychosocial and physical summary scores, was dichotomised (parents’ and children’s self-reports in each country). Values above the medians were defined as high rated HRQOL and below the medians as low values, respectively (Table 7). A binary logistic regression model was created to identify factors influencing generic HRQOL in the study population. The sample size (*n* = 58) justified two independent variables being introduced in each model. The independent variables (self-reports and proxy versions) were: gender, age groups, diagnostic groups, treatment modalities, time since diagnosis, and parents’ employment. In a logistic regression analysis, the HRQOL rating of children aged five to 12 years of age in Sweden significantly predicted generic psychosocial (ENTER, OR 7.5, *p* = 0.007) and physical health (ENTER, OR 6.2, *p* = 0.011). None of the other models were significant.

**Table 7:** PedsQL™, Generic Core scale, including subscales, in Sweden and Argentina

Modules	Generic Core Scale (total score)		Psychosocial health summary		Physical health summary	
	Children	Parents	Children	Parents	Children	Parents
Median	64.7	58.0	68.3	63.3	62.5	53.1
Mean (sd)	65.4(16.9)	60.9(17.2)	68.4(15.1)	64.4(14.4)	60.4(23.7)	55.7(27.1)

*sd* standard deviation

## Discussion

The present study evaluated HRQOL in children and adolescents with cancer in Argentina and Sweden. The psychometric instrument, PedsQL™, was distributed and the response rate was high. The gender distribution of malignant diseases was in line with epidemiological reports from the two countries [4, 5], i.e. more boys than girls were affected. The leukaemia group was dominant and few children with CNS tumours participated. The distribution of gender, diagnoses, the mean age and mean time on treatment were in line with previous research validating the PedsQL™ instrument [11]. The majority of the proxy versions were answered by mothers, i.e. there was the same parental gender distribution as previously observed [10]. Generally low estimations of HRQOL were found in the patients’ self-reports and corresponding parent proxy versions in both countries. When comparing the two countries and corresponding age groups, teenagers in Argentina reported higher levels of generic HRQOL and school-aged children had more cancer-related problems. Patient and proxy versions in Argentina revealed fewer problems regarding fatigue among male participants, adolescents, and patients with solid tumours. More cancer-related problems and a greater fatigue load were generated by multimodality treatment. Within Sweden, the multidimensional fatigue challenge was most prominent among adolescents and in the group of solid tumours. Teenagers reported lower HRQOL (generic and cancer-specific) compared to the school-age group. Good agreement was found between children and proxy versions in every module in Sweden and in the fatigue and generic physical health scales in Argentina. In Sweden, the central role of generic psychosocial and physical health was predicted by children being in the school-age group.

Psychological distress is a well-known factor influencing QOL [12, 15]. The low HRQOL rating in comparison with healthy samples was an expected result due to the cancer experience [10, 11, 18, 20, 21, 30]. Lower socioeconomic status may influence HRQOL in children with cancer, but the results are not conclusive [15, 30]. In this study, we analysed the level of parental education and determined whether the parents were employed or unemployed, but we did not consider their socioeconomic status. In Argentina, in comparison with Sweden, more parents were unemployed, but they still reported fewer problems of cancer-related fatigue; facts which were also reported by their children. In Sweden, children whose mothers had post-secondary education stated fewer cancer-related problems [30]. There are obvious differences between the countries. In Sweden, when a child is seriously ill both parents can be off work at the same time, paid by social insurance benefit days. Such an opportunity is lacking in Argentina. Your burden is great if you are unemployed while a parent to a child with cancer, and these circumstances will probably cause a high level of distress [35], and increase your level of fatigue, although this was not conveyed in the current study. However, if parents and children consider fatigue as an inevitable consequence of treatment they may not report the severity of the problem, nor emphasize approaches to reduce it [36].

Age-related differences were observed between countries, and teenagers emerged as a vulnerable group, especially in Sweden. Adolescents have the maturity to realise the magnitude of the problem of being diagnosed with a life-threatening disease. Independency from parents, the personal identity process, and social autonomy are some of the challenges in this period in life [37]. The Swedish National Board of Health and Welfare presented a report in May 2013 describing deterioration in young people’s mental health in recent decades, especially in girls [38]. Several HRQOL studies including paediatric cancer patients [20, 21, 29] and childhood cancer survivors [15, 39] have shown the same gender differences. Few studies have presented the opposite [20]. In the present study boys reported lower HRQOL in several sections, all though not statistically significant. Our results may be related to the small sample size and selection bias or may indicate that girls do better during cancer treatment [20].

Culture may be defined as shared values in a group [40]. According to the World Values Survey, 2015, Sweden is a country with strong secular-rational and self-expression values, while Argentina has more traditional values [41]. In Latino culture the role of the family is powerful, with strong affiliation and cooperation (“familismo”) [40]. Cultural aspects of family constellations may influence how teenagers express and communicate their symptoms and feelings. Good family cohesion may create a trustful environment, which might prevent psychological distress and symptoms of loneliness and exclusion. On the other hand, young people may choose to give high HRQOL ratings with the goal of protecting their parents from knowing their deepest thoughts and feelings [16]. This choice may be a consequence of a culture characterised by low levels of openness to discussing serious issues regarding life-threatening disorders and the risk of dying. When collecting dyadic information worries and communication concerns may be disclosed regarding the child’s physical and psychosocial status [32]. This meaningful understanding may also define agreement and disagreement in different aspects of HRQL in childhood. However, poor agreement in patient and proxy versions is often reported for psychosocial HRQOL domains, unlike physical impairments [14, 42], but was not the case in the Swedish population. We speculate that these results may be related to a cultural openness to communicating about demanding topics. In Western psychosocial care, when a child is diagnosed with cancer truthfulness is essential. Parents are encouraged to let their child participate in decision-making and to have open and age-appropriate communication [40]. This directness can reduce the levels of distress concerning the disease and may pave the way for parents to keep on communicating with their child regarding psychosocial issues [43]. In many cultures, nondisclosure of malignant disorders to a child is tolerable [40, 44]. In Sweden, every study participant was informed about his/her diagnosis but in Argentina some of the patients were not fully informed about their disease in accordance with their parents’ wishes (MM). Still, we do not know for sure the reasons for the differences observed between countries, as we have not used valid instruments or performed interviews to assess dyadic communication. However, based on the parental perspective, we would argue that it may also be a greater psychosocial challenge for parents to comfort and support teenagers compared to children of lower ages. Toddlers are very dependent on a secure attachment to their parents and daily routines, but due to aspects of maturity teenagers have a more complex life situation [12]. If health professionals support the parents in their parental role it may have an impact on the HRQOL of their little child [22]. To reach the adolescents, there is also a need for communication skills, knowledge covering developmental and age-related differences, and cultural competence [12].

Compared to the teenagers, the group of school-age children had consistently higher HRQOL ratings and in Sweden, their rating influenced both their general physical and psychosocial health. This may indicate that school-age children, despite the disease, do fairly well. The developmental period of around five to 12 years is a time when children put their faith in rules and schedules and may be distressed at not having an identifiable cause of their disease [12]. Being in school creates routines and has psychosocial advantages, such as interacting with school mates and teachers. Children in Sweden are encouraged to attend school as much as possible during cancer treatment, and they have reported better HRQOL if they do so frequently [29]. Consultant nurses, in collaboration with the families, visit schools to provide the personnel and classmates with information. This is in line with the role of openness related to the disease and its consequences. In Argentina, many children have private home-bound teaching during the period of illness, which may change the child’s previous habits and limit social interactions with peers and negatively influence QOL [45].

Children and parents in both countries confirmed and highlighted the major problem with fatigue and that multiple treatments generate more challenges [37]. Paediatric oncology patients all over the world have previously judged fatigue to be the most troublesome disorder [11, 14, 46–48]. The disease-specific rating and the multidimensional fatigue rating often have several common determinants [11]. Physical impairments and symptoms of fatigue are factors known to reduce the HRQOL, even in a long-term perspective [39]. In the present study, patients with solid tumours appeared as a vulnerable group, but not children with CNS tumours, despite the fact that both these diagnostic groups have multimodality therapy regimes. The explanation is probably the small study population of patients with CNS tumours. The cultural differences regarding fatigue challenges, with generally fewer cancer-related problems in Argentina, are unclear, but may be related to the above described diverse cultural beliefs. We speculate that when you live in a community where you are encouraged to participate in social activities, the discrepancy between having a severe illness and being in a state of health may be more obvious. In environments with reduced social interactions this exhaustion may not be as salient.

The study sample represented children and adolescents with cancer in two different countries and continents. The participants had diverse ethnic and cultural backgrounds. Our ambition was to recognise the child’s own perspective on health, illness and treatment [10], but also to determine the relationship between children and proxy ratings [14]. To establish cross-cultural trustworthiness, one of the researchers (ES) met every participating child and parent.

We are aware of several limitations related to this study design and conclusions must be drawn with caution. The small sample size and heterogeneous population restricted our ability to perform sub-group analyses [15], and lacked power to evaluate other factors that are known to influence HRQOL in children with cancer, e.g. toxicity and side effects of oncological treatment and psychosocial factors affecting family life. The cross-sectional design with a single assessment limited the ability to comment on the child’s entire experience of the cancer trajectory. Four out of 62 parents, i.e. two couples in each country, answered the proxy versions together. This is a bias as parents may have influenced each other in the estimation of their child’s HRQOL.

## Conclusions

The current study highlights the importance of performing comparative studies of HRQOL of young individuals with malignant disorders from different cultural backgrounds. There is a lack of evidence in the literature about whether perception of HRQOL differs across countries [32]. There is also a dearth of information regarding the association between agreement and communication. Investigations are needed to determine more satisfactorily how agreement is affected by the child’s health [14]. Healthcare professionals need communication skills, cultural competence, and knowledge to capture individual HRQOL challenges in paediatric oncology. To deepen and increase the knowledge regarding the role of culture and communication we propose longitudinal studies with interventions and interviews. The interview studies could be designed as patient/clinician-based, but also interview-based with patients and their parents.

## Abbreviations

CNS: Central nervous system
HRQOL: Health-related quality of life
PedsQL™: Pediatric Quality of Life Inventory™
QOL: Quality of life
